# A comparative pharmacological investigation of three samples of '*Guduchi ghrita*' for adaptogenic activity against forced swimming induced gastric ulceration and hematological changes in albino rats

**DOI:** 10.4103/0974-7788.64399

**Published:** 2010

**Authors:** Shriram S. Savrikar, Vilas Dole, B. Ravishankar, Vinay J. Shukla

**Affiliations:** *R. A. Podar Medical College (Ayurved), Worli, Mumbai, India*; 1 *Department of Rasashastra, Tilak Ayurved College, Rasta Peth, Pune, Maharashtra, India*; 2 *Institute of Postgraduate Training and Research in Ayurved, Gujarat Ayurved University, Jamnagar, Gujarat, India*

**Keywords:** Ayurveda, adaptogenic activity, '*Guduchi ghrita*', '*rasaayana*', *Tinospora cordifolia* (Willd.) Miers

## Abstract

This study was undertaken to investigate the impact of formulation factors and adjuvants on the expression of biological activity of *Tinospora cordifolia* (Willd.) Miers. The adaptogenic effect of three samples of *Guduchi ghrita*, prepared using plain *ghee* (clarified butter) obtained from three different sources was studied in albino rats and compared with expressed juice of stem of *Guduchi*. The test preparations were evaluated against forced–swimming induced hypothermia, gastric ulceration and changes in the hematological parameters. The test drug given in the form of '*ghrita*' produced better effect in comparison to the expressed juice. Among the three '*ghrita*' preparations evaluated, only the '*Solapur Guduchi ghrita*' (SGG) was found to produce significant inhibition of stress hypothermia and gastric ulceration. The other two preparations '*Nanded Guduchi ghrita*' (NGG), and '*Wardha Guduchi ghrita*' (WGG) could produce only a marginal effect. In hematological parameters '*Guduchi*' juice produced better reversal of the stress-induced changes in comparison to the test '*ghrita*' preparations. The present study provides evidence highlighting the importance of formulation factors for the expression of biological activity.

## INTRODUCTION

'*Ghrita*',also known as clarified butter, is a traditional adjuvant/ vehicle described in Ayurveda.[1] Processing of '*ghrita*' with plant material is renowned for enhancing the therapeutic efficacy of the plant ingredients. In this reference, a specific word '*Sahasraveerya*,' depicting infinite potency or power of '*ghrita*,' is used by Charaka. The '*ghrita*' is believed to be made more potent through its processing with substances possessing different properties. More interestingly, '*Ghrita*' also possesses the property of '*yogavaahitwa*' by way of which it enhances the properties of substances with which it is processed.[1] *Tinospora cordifolia* (Willd.) Miers., (Menispermaceae) is described in Ayurveda as a '*rasaayana*'[2] and recent studies have demonstrated significant immunomodulation activity with this plant.[3,4] The plant is routinely used in the treatment of chronic fever.[5] Our earlier studies have demonstrated the antipyretic effect produced by '*Guduchi ghrita*', is significantly superior to the antipyretic effect observed in '*Guduchi*' juice-administered group.[6] The antipyretic effect observed in '*Guduchi*' juice-administered group was comparatively faster in appearance but was short lived. It appeared late but sustained for a longer period up to 12 hours, in all the three '*Guduchi ghrita*' groups. The present study was conducted to evaluate the adaptogenic activity and hematologic effects of different formulations of *Tinospora cordifolia* in *ghrita* (ghee) obtained from various sources and the expressed juice of the stem of *Guduchi*.

## MATERIALS AND METHODS

### Plant material

Mature stems of *Tinospora cordifolia* (Willd.) Miers were collected during winter (November 2005), as this is the suitable season for collection of plants,[7] Collection was made from wild sources. The plant was identified and authenticated by the concerned authority in the Department of Pharmacognosy of the Institute, and the voucher specimen was deposited with the Pharmacognosy laboratory.

### '*Ghrita*', (clarified butter or butter oil)

Three samples of '*ghrita*', clarified butter or ghee prepared in the traditional way from cow's milk, were obtained from three different places namely Wardha, Nanded and Solapur and were used as the base materials for preparing '*Guduchi ghrita*'.

### Preparation of '*Guduchi ghrita*'

Three samples of '*Guduchi ghrita*' each from 1 kg of plain ghee were prepared by one person at one place for this study in accordance with the formulation prescribed by 'Vaagbhata and explained by Indu'.[8] The proportion of '*kalka*' i.e. paste of '*Guduchi*', '*ghrita*' and '*swarasa*' of '*Guduchi*' was 1:4:16 respectively as prescribed by Indu.[8] The stem of '*Guduchi*' is hard in consistency and does not yield '*swarasa*' in adequate quantity by the method of crushing and squeezing the material. Hence the method described by Sharngadhara*[9] was used to obtain '*Guduchi swarasa*'. The stem of '*Guduchi*' was crushed and overnight soaked in double the quantity of water. The contents were then filtered through muslin cloth. The filtrate so obtained was used as '*Guduchi swarasa*' (Expressed juice). All the ingredients were placed in a container, and were heated on a gas stove to evaporate aqueous part. The contents were stirred regularly to avoid burning. Heating was stopped when the '*ghrita*' passed all the tests described in the text, such as disappearance of froth, formation of a wick, burning of '*ghrita*' without crackling noise etc.[10] The contents were then filtered through a muslin cloth. The filtrate i.e. '*Guduchi ghrita*' was collected in a clean autoclaved glass bottle and stored in a cool place. The test '*Guduchi ghritas*' were named after the places from which the plain '*ghrita*' samples were obtained, as Nanded *Guduchi Ghrita* (NGG), Wardha *Guduchi Ghrita* (WGG) and Solapur *Guduchi Ghrita* (SGG). The three samples of plain '*ghrita*' used and the three samples of test formulations were subjected to preliminary physical and chemical investigations. Noted observations are shown in Tables 4‐6. '*Guduchi*' sample was tested for bitterness value. Macro and microscopic studies of the sample were also carried out. High performance thin layer chromatograph profile of the plant material used, and the three final formulation products, was recorded.[11] Since the focus of this study was on assessing the possible influence of use of '*ghritas*', sourced from different places, the same plant material was used for all the preparations.

### Animals

Charles Foster strain albino rats of either sex weighing between 140 and 200g, procured from the institute's animal house were used. They were maintained on 'Amrut' brand rat/mice pellets and exposed to ambient temperature, humidity and natural day and night cycles. All the experiments were carried out between 8 am and 12 noon. The studies were conducted in conformity with the guidelines of the Institutional Animal Ethics Committee after obtaining its permission.

### Dose selection

The human dose of '*Guduchi ghrita*' as practiced traditionally is 10 g per person. (The dose of '*ghrita*' preparations as prescribed by Ayurvedic Formulary of India (AFI) is 12 g. However 10 g dose was taken for this study as an average dose.)[12] Accordingly, the animal dose was calculated as 900 mg/kg body weight for rats on the basis of body surface area ratio.[13]

### Evaluation for adaptogenic activity

The effect of test drugs was evaluated on forced swimming stress induced hypothermia and gastric ulceration in rats following the method described by Kulkarni and Goel.[14] Thirty six rats of either sex were divided into six groups of 6 animals each. To each of the groups the following treatment was given i) water *ad libitum* ii) '*Guduchi swarasa*' (expressed juice obtained from the stem of *Tinospora cordifolia* in the dose of 1.8mL/kg equivalent to a 20mL human dose), and iii) WGG 900 mg/kg body weight orally for 7 days, iv) NGG 900 mg/kg body weight orally for 7 days, v) SGG 900 mg/kg body weight orally for 7 days, vi) Plain '*ghrita*' (pooled '*ghrita*' prepared by mixing the three plain '*ghritas*' obtained from three places.

The test drugs and vehicles were orally administered daily on 7 consecutive days. On the eighth day, following fasting of rats for 48 hours, the rats were forced to swim in a water column with a height of 18 cm at 30°C in a plastic container measuring 25cm in height and 10 cm in diameter. Baseline recording of rectal temperature was made with a Century's telethermometer. At the end of 30 minutes, the rats were taken out from the water and rectal temperature was recorded again. Following this, the rats were placed back in the water in the plastic container and the forced swimming was continued for a total duration of 12 hours. At the end of 12 hours the rats were taken out and dried with the help of a hair drier. They were then sacrificed by cervical dislocation. Around 4 mL of blood was collected in a vial containing anti-coagulant, Ethylene Diamine Tetra Acetate (EDTA). The samples were used for estimation of hematological parameters. Hematological parameters were estimated with the help of an auto cell counter (MS-9 of Melet Schloesing Lab, France), which along with RBC counts, additionally gives size based quantification of RBC (micro and macro). After careful laparotomy, the stomach was excised out by opening along the greater curvature. It was washed gently with tap water. The inner surface of the stomach was carefully observed with a magnifying lens for the number of ulcers and severity of ulceration in glandular area as well as in lumen. [14] The severity of the ulceration was graded as under:

0‐No visible ulcer
1‐Approximate ulcerated area up to 1 mm. in diameter
2‐Approximate ulcerated area up to 2 mm. in diameter
3‐Approximate ulcerated area up to 3 mm. in diameter
4‐Approximate ulcerated area more than 3 mm. in diameter
5‐Perforation of the gastric wall

Severity of the ulceration and the number of ulcers in each rat stomach were recorded for calculating ulcer index.

### Statistical analysis

The data are presented as Mean ± Standard Error of Mean (SEM) for 6 animals per group. Comparison between control and test drug administered groups was made by one way ANOVA followed by Dunnet's multiple 't' test or by unpaired 't' test. *P*< 0.05 was considered to be significant. In case of Ulcer index Mann Whitney U test was employed. The analysis was carried out using Sigma Stat version 3.1 and Graph pad 3.

## RESULTS

### Stress-induced hypothermia and gastric ulceration

The data pertaining to the effect of different types of treatments on forced-swimming-stress-induced hypothermia are shown in Table 1. The difference between the temperatures was noted and converted to percentage decrease in temperature.

**Table 1:** Effect of different preparations of *Tinospora cordifolia* stem on forced-swimming stress-induced hypothermia and gastric ulceration in albino rats

Group	% decrease rectal temp	Ulcer index
Water-control	16.80 ±1.07	6.38 ± 0.35
Plain '*ghrita*'-control^b^ 900 mg/kg	12.18 ± 0.71**@	4.60 ± 1.20
*Guduchi* juice^b^ 1.8 ml/kg	13.95 ± 0.54@	11.60 ± 3.24
WGG^b^ 900 mg/kg	13.09 ± 0.96 *@	8.62 ± 2.89
NGG^b^ 900 mg/kg	12.98 ± 0.56 *@	5.00 ± 2.32
SGG^b^ 900 mg/kg	06.45 ± 1.06***	2.75 ± 0.94#

Data is expressed as Mean± SEM.

* *P*<0.05 and

*** *P*<0.001 as compared to water control,

@ *P*<0.001 as compared to SGG using ANOVA.

# *P*<0.05 as compared to water control using Mann Whitney U test,

b These groups are subjected to forced swimming stress. WGG- Wardha *Guduchi ghrita*, NGG- Nanded *Guduchi ghrita*, SGG- Solapur *Guduchi ghrita*

Marked hypothermia was observed in water control rats subjected to forced-swimming stress. In pooled plain '*ghrita*'-, test *ghritas*, and '*Guduchi swarasa*'-administered groups an apparent decrease in hypothermia was observed in comparison to water control. However, the decrease observed in pooled plain *ghrita* and test *ghritas* was found to be statistically significant. When test ghritas were compared to pooled plain '*ghrita*' control group, the decrease observed in SGG was also found to be significant. However, the observed difference in other groups was non-significant.

Moderate–to-severe ulceration in the stomach was observed in control rats subjected to forced-swimming stress. In pooled plain '*ghrita*'-administered group, the severity of ulceration was 27.8% less in comparison to normal control. Severity of ulceration increased slightly in WGG- and moderately in NGG administered group. However, in SGG-administered group, significant decrease was observed in comparison to water control. When the data of SGG treated group was compared with pooled plain *ghrita* control group though 40% decrease in severity was noted, the difference was found to be statistically non-significant. Surprisingly in '*Guduchi swarasa*' administered group, the severity was more than doubled in comparison to pooled plain '*ghrita*' group. Because of the variation of the data and large SEM, the observed difference was found to be non-significant.

### Hematological parameters

#### WBC related parameters

The data related to the effect of test preparations on stress induced changes in WBC related parameters can be seen in Table 2.

**Table 2:** Effect of different preparations of *Tinospora cordifolia* stem on forced-swimming-stressinduced changes in WBC related parameters in albino rats

Groups	WBC (103/ µl)	Lymphocytes		Granulocytes		Monocytes	
		(103/µl)	±	(103/µl)	±	(103/µl)	±
Control without stress	2.38 ± 0.39	2.16 ± 0.34	83.70 ± 0.81	0.28 ± 0.04	12.44 ± 0.94	0.10 ± 0.00	3.86 ± 0.16
Water control^b^ 1.8 ml/kg	1.12 ± 0.20	1.72 ± 0.32	92.00 ± 0.96@@	0.12 ± 0.04	5.02 ± 1.11@@	0.04 ± 0.02	2.24 ± 0.30@
Plain *ghrita* 900 mg/kg^b^	1.38 ± 0.32	1.67 ± 0.51	93.35 ± 1.05@@@	0.10 ± 0.03@	4.72 ± 0.73@@	0.03 ± 0.02	1.95 ± 0.30@
*Guduchi* juice^b^	1.92 ± 0.36	1.67 ± 0.32	87.13 ± 1.44£#	0.18 ± 0.05	9.95 ± 1.38#	0.10 ± 0.03	2.92 ± 0.15
WGG^b^ 900 mg/kg	1.76 ± 0.19#	1.62 ± 0.18	91.16 ± 1.16@	0.12 ± 0.06	6.50 ± 1.08@	0.03 ± 0.02	2.04 ± 0.69@
NGG^b^ 900 mg/kg	1.83 ± 0.17#	1.62 ± 0.16	89.50 ± 2.64	0.14 ± 0.05	8.24 ± 2.24@@	0.02 ± 0.02	2.16 ± 0.34@
SGGb 900 mg/kg	1.80 ± 0.56*#	1.28 ± 0.31	93.40 ± 0.92@@@	0.05 ± 0.02@	4.73 ± 0.62	0.05 ± 0.02	1.87 ± 0.32@@

Data is expressed as Mean± SEM.

* *P*<0.05 as compared to plain *ghrita*,

@ *P*<0.05,

@@ *P*<0.001 and

@@@ *P*<0.05 as compared to control without stress,

£ *P*<0.05 as compared to SGG using ANOVA.

# *P*<0.05 as compared to stress water control using Unpaired 't' test.

b These groups are subjected to forced swimming stress

Rats subjected to forced-swimming stress showed marked decrease (52.94%) in total WBC count in comparison to non-stressed control rats. In pooled plain '*ghrita*' administered group, the decrease in count was marginally less in comparison to stress control group. In test preparation-administered groups, the observed decrease was further less in comparison to the stress control group and was statistically significant employing unpaired 't' test. This difference was not reflected when ANOVA test was applied, however this test demonstrated significant difference between pooled plain '*ghrita*' administered group and SGG treated group.

Forced-swimming stress was found to decrease lymphocyte, granulocyte and monocyte counts in comparison to normal control rats, which was statistically significant in pooled plain '*ghrita*' and SGG treated group. The stress-induced decrease observed in lymphocyte count was not reversed by treatment with either pooled plain '*ghrita*' or test '*Guduchi ghrita*' preparations. Weak reversal of the stress-induced decrease in granulocyte count was observed in '*Guduchi*' juice and NGG-administered groups. However, the reversal did not reach statistically significant level. Other preparations had no effect on this parameter. Moderate reversal of stress-induced decrease in monocyte count was observed in '*Guduchi*' juice administered group but it was statistically non-significant. No reversal could be observed in other test preparations-administered groups.

Though absolute count of lymphocyte was found to be decreased (probably due to decrease in the total WBC count) in stressed animals, in comparison to normal control when the data were presented as percentages, significant increase in lymphocyte percentage and significant decrease in granulocyte and monocyte percentages were observed in stress control rats in comparison to normal control rats. The elevated lymphocyte percentage due to stress was not significantly reversed by any of the treatment mode employed (ANOVA). However, the *guduchi juice* treated group showed significant decrease in this elevation against stress water control when compared using unpaired 't' test.

The stress-induced decrease in granulocyte percentage was antagonized by the administration of '*Guduchi*' juice, which did not reach statistically significant level (ANOVA). However, when compared using unpaired 't' test, it showed significant increase against stress water control. Similar non-significant reversal was observed in NGG and WGG administered groups.

In case of monocyte percentage, *Guduchi* juice reversed the stress induced decrease, which was not significant statistically.

#### RBC related parameters [Table 3]

Mean Corpuscular Volume (MCV) and Hematoctrit (HCT)% were found to be apparently increased in stress water control in comparison to normal water control groups. Although, unpaired 't' test showed that the observed increase is significant, ANOVA test did not show this difference.

The apparent elevation observed in MCV was found to be significantly reversed in the pooled plain *ghrita* group, '*Guduchi*' juice and NGG administered groups. Elevation of HCT (%) was found to be significantly reversed in the '*Guduchi*' juice and SGG administered groups. The reversal observed in pooled *ghrita*, NGG and WGG administered group was found not significant using ANOVA although when unpaired 't' test was applied the observed difference in means was significant in the pooled plain *ghrita* and NGG groups as well.

There was increase in MCHC level in rats subjected to forced-swimming stress as compared to normal rats. There was no significant effect on this parameter in any of the treated groups.

Micro RBC formation was not observed in any of the groups studied. An increase in macro RBC count was observed in stress water control in comparison to normal water control. The increase observed in macro RBC count was found to be unaffected in pooled plain '*ghrita*', WGG and SGG administered groups. The moderate reversal observed in NGG-administered and '*Guduchi*' juice-administered groups was statistically non-significant.

The data related to physico-chemical investigation of *ghrita* is summarized in Tables 4‐6. The physico-chemical tests were mainly performed to ascertain whether the ghee is adulterated or not. The specific gravity should be around 0.910 to 0.920; the refractive index (at 40°C), 1.453 to 1.456,[15] iodine value, 26-38; and saponification value, 220-232.[16] Since the values observed were within this range, it can be suggested that the samples used were free from gross adulteration. In SGG the cholesterol content of the unsaponifiable matter was higher in comparison to the plain ghee while it was less in WGG and NGG. The triglyceride content was less in SGG and WGG and more in NGG '*Guduchi ghrita*'.

**Table 3:** Effect of different preparations of *Tinospora cordifolia* stem on forced-swimming-stressinduced changes in mean cell volume, hematocrit and mean corpuscular hemoglobin concentration in albino rats

Group	MCV (fL)	HCT (%)	MCHC (g/dL)	RBC count 106/µL	Macro RBC (%)
Control without stress	73.40 ± 0.53	53.94 ± 3.59	8.45 ± 0.37	7.37 ± 0.53	0.93 ± 0.05
Water - control^b^	78.58 ± 0.43	67.44 ± 1.68	16.38 ± 0.07	8.67 ± 0.26	1.11 ± 0.05
Plain '*ghrita*' 900 mg/kg^b^	61.96 ± 4.77**@@@	53.76 ± 5.57+	18.83 ± 0.40	7.99 ± 0.79	1.12 ± 0.14
'*Guduchi*' juice 1.8 mL/ kg^b^	67.84 ± 0.98@@§	47.34 ± 1.25@^+++^	18.48 ± 0.35	6.02 ± 0.89	0.83 ± 0.17
WGG 900 mg/kg	74.04 ± 1.21££	58.84 ± 5.20	19.28 ± 2.05	6.99 ± 0.24	1.06 ± 0.14
NGG 900 mg/kg^b^	66.63 ± 0.83@@§	48.96 ± 2.40^+++^	18.10 ± 0.59	7.97 ± 0.73	0.76 ± 0.17
SGG 900 mg/kg^b^	77.06 ± 0.98££	46.65 ± 6.98@+	16.98 ±0.26	7.36 ± 0.37	1.15 ± 0.15

Data is expressed as Mean± SEM.

** *P*<0.01 as compared to control without stress,

@ *P*<0.05,

@@ *P*<0.01 and

@@@ *P*<0.001as compared to stress water control,

££ *P*<0.01 as compared to plain *ghrita*,

§ *P*<0.05 as compared to SGG using ANOVA.

+ *P*<0.05 and

++ *P*<0.001 as compared to stress water control using Unpaired 't' test.

b These groups are subjected to forced swimming stress, MCV - mean cell volume, HCT - hematocrit, MCHC - mean corpuscular hemoglobin concentration

**Table 4:** Organoleptic characters SGG, NGG, WGG and plain ghee

	SGG	NGG	WGG	Plain ghee
Consistency	Semisolid	Semisolid	Semisolid	Semisolid
Color	Green	Yellowish	Green	Yellowish
		Green		
Odor	Characteristic	Characteristic	Characteristic	Characteristic
Taste	Bitter	Bitter	Bitter	Characteristic

WGG- Wardha Guduchi ghrita, NGG- Nanded Guduchi ghrita, SGG- Solapur Guduchi ghrita

**Table 5:** Consolidated data of different parameters assessed with the test samples

Sample	Specifi c gravity at 40°C	Acid value	Free fatty acids (FFA)% w/w	Saponifi cation value	Iodine value
Plain '*ghrita*'	0.910	2.36	1.19	216.6	33.95
WGG	0.911	2.84	1.42	226.2	33.72
NGG	0.910	1.84	0.92	220.5	28.77
SGG	0.911	2.70	1.36	221.9	33.76

**Table 6:** Cholesterol, unsaponifi able matter and triglyceride content in different test samples

Sample	Unsaponifi able Matter % W/W	Cholesterol mg/100 g	Triglyceride mg/mL
Plain ghee	0.36	240.6	14.64
SGG	0.50	256.8	10.07
WGG	0.54	207.5	12.81
NGG	0.35	222.4	17.08

## DISCUSSION

The science of drug formulation, termed as '*Bhaishajya kalpana*' in Ayurveda, has an important place in Ayurvedic therapeutics. Clear-cut guidelines, such as parts of plants to be used, processes to be followed for formulations, along with indications regarding disease- and patient-specific formulations, have been prescribed in ancient Ayurvedic texts. [17] It is believed that the therapeutic efficacy of the drug is significantly affected by various factors related to the nature of ingredients in the formulation. Similarly it is also possible that adjuvant/vehicles and other excipients used in the formulation may have an impact on expression of biological activity.

Chemically, '*ghrita*' is 99.5% milk fat. It is a complex lipid of glycerides, free fatty acids, phospholipids, sterols, sterol esters, fat-soluble vitamins, tocopherol, carbonyls, hydrocarbons, carotenoids, small amounts of charred casein, traces of minerals like calcium, phosphorus, iron, copper etc. Ayurveda describes that '*Ghrita*', is a useful adjuvant/ vehicle. Administering the plant material incorporated in a *ghrita* is known to enhance the therapeutic efficacy of the plant ingredient. Characteristics of '*ghrita*' suitable for processing have also been described in ancient Ayurvedic classics.[18] Besides acting as a vehicle and an adjuvant, *ghrita* has been described to exert therapeutic effects like '*rasaayana*' (adaptogenic activity). Moreover, '*ghrita*' has been described to absorb the properties of the material with which it is processed ('*sanskaraanuvartana*').[19]

Our study indicates that administration of '*Guduchi*' in the form of ' *ghrita*' exerts better adaptogenic activity compared to '*Guduchi swarasa*' (expressed juice) in the forced-swimming stress- induced hypothermia and gastric ulceration model. In fact, increased ulceration was observed in the group that received '*Guduchi*'-expressed juice alone. Interestingly adaptogenic activity observed was different in the three formulations. SGG was effective in providing protection against hypothermia as well as production of gastric ulcers, while WGG and NGG failed to produce a significant effect. The reason behind the observed difference among the three test formulations is not clear from our results. It is to be noted that the same type of expressed juice from the same batch was used for the preparation of '*Guduchi ghrita*'; only the plain '*ghrita*' used was different. This clearly indicates that the quality of plain '*ghrita*' used can have important influence on the activity expressed. The chemical- or formulation-related basis for the observed difference needs to be probed. It is very important to obtain such information considering that this difference will have serious implications in clinical settings.

In the present study, significant elevation in RBC count, macro RBC percentage, MCV, HCT% and significant decrease in MCHC and Hb were observed in stress control group. This contradictory data may be due to increase in the number of immature RBC leading to increased RBC count without concomitant increase in Hb%. It has been observed that kidney plays an important role in the regulation of formation of RBC through formation of erythropoietin. Hypoxia in the kidney is the major stimulant for the increased formation of RBC through secretion of erythropoietin.[18] It can be suggested that factors like catecholamines and glucocorticoids released as a result of HPA activation through stress response may be interfering with the blood supply to the kidney. This may be causing hypoxia, leading to increased formation of erythropoietin and hence increased RBC count. This may be the reason for the observed elevation in RBC count in forced-swimming-stress control animals. This stress response was significantly reversed by the administration of WGG, SGG and '*Guduchi*' juice, whereas NGG was less effective. It is to be noted that the activity profile of the test formulations is different in different sets of parameters. This indicates possible involvement of different factors and existence of difference in the test formulations' ability to modulate them. However, one feature that stands out is that SGG was able to produce anti-stress activity against majority of the parameters recorded. This indicates that the SGG has special attributes that potentiate the adaptogenic activity of '*Guduchi*' juice. The exact nature of the potentiation observed in this study requires further elucidation. The data generated in this study can be taken as an example of the importance of different formulation factors in the expression of biological activity.
